# Acupuncture in circadian rhythm sleep–wake disorders and its potential neurochemical mechanisms

**DOI:** 10.3389/fnins.2024.1346635

**Published:** 2024-01-22

**Authors:** Junmei Wu, Zhengyu Zhao

**Affiliations:** Acupuncture and Tuina School, Chengdu University of Traditional Chinese Medicine, Chengdu, China

**Keywords:** acupuncture, circadian rhythm sleep–wake disorders, biological clock, neurochemical mechanisms, review

## Abstract

Circadian rhythm sleep–wake disorders (CRSWDs) are becoming increasingly common in modern societies due to lifestyle changes. The detrimental effects of CRSWDs on sleep and psychological health have attracted considerable attention recently. Alternative remedies for the treatment of CRSWDs have also gained attention in recent years owing to the limitations of medications. Several *in vivo* and clinical investigations have shown that acupuncture, one of the most important components of traditional Chinese medicine (TCM), has been shown to modulate sleep-related circadian rhythms. Owing to the lack of research on the mechanism and effectiveness of acupuncture in treating CRSWDs, clinical applications of acupuncture have not gained popularity. This paper reviews the acupuncture methods, acupoint selection, and biochemical indicators supplied by *in vivo* and clinical studies to explore the effectiveness of acupuncture, and summarizes the circadian rhythm mechanisms and the acupuncture characteristics on circadian rhythm. The neurochemical mechanisms linked to acupuncture in treating CRSWDs are also outlined from the perspective of the central and peripheral biological clocks. Lastly, the inadequacy of previous studies on CRSWDs and conflicting results regarding acupuncture are explored and future research directions are envisioned.

## 1 Introduction

Circadian rhythm refers to the autonomous regulation of the organism’s periodic oscillation of the cycle for approximately 24 h. One of the most noticeable circadian rhythms in the human body is the sleep–wake cycle. In recent years, research on insomnia has focused mostly on circadian rhythm sleep–wake disorders (CRSWDs). The precise prevalence of CRSWDs is unknown since it varies depending on the type of population being assessed. An early study that included 10,000 Norwegian people aged between 18 and 67 years discovered that the prevalence was 0.17%, with an average age at onset of 15.4 years (Schrader et al., 1993). A recent population study conducted on adults in New Zealand found that the prevalence of sleep delay types ranges from 1.5 to 8.9% (Paine et al., 2014). However, the actual prevalence of CRSWDs is underestimated because CRSWDs are frequently mistaken for other sleep disorders. According to some estimations, up to 10% of individuals with sleep disturbances are thought to have CRSWDs (Barion and Zee, 2007).

CRSWDs are most frequently characterized by difficulties in initiating and maintaining sleep and excessive sleepiness. Based on the standards provided by the International Classification of Sleep Disorders (ICSD-III) (Sateia, 2014), CRSWDs can be generally divided into two types: sleep disorders caused by endogenous circadian rhythm system changes, such as delayed sleep-phase disorder (DSPD), advanced sleep-phase disorder (ASPD), non-24-h sleep–wake syndrome, and irregular sleep–wake rhythm; or sleep disorders caused by social/work schedules, such as jetlag and shiftwork. Current medical interventions for CRSWDs are mainly focused on pharmacologics, including benzodiazepines (BZDs), melatonin (MT) receptor agonists, and antidepressants (Atkin et al., 2018; Everitt et al., 2018). However, some of these interventions are not well tolerated or safe, and their prolonged use can result in mental and physical dependence (De Crescenzo et al., 2022). Therefore, safe and reliable non-pharmacological treatments are needed.

One of the major non-pharmacological treatments for people with sleep disorders is acupuncture, which has been extensively used to treat CRSWDs. Acupuncture is a method of manipulating the qi and blood, balancing yin and yang, and regulating the disharmony of zang and fu organs, by inserting thin metal needles into specific acupoints on the body (Kaptchuk, 2002; Ernst, 2006). Alhough, many clinical trials or mechanistic studies on acupuncture treatment for CRSWDs have been published, evidence of the effectiveness of acupuncture has not been thoroughly compiled. Therefore, this article reviews the acupuncture method, acupoint selection, and biochemical indicators to investigate the effectiveness of acupuncture based on clinical trials and *in vivo* studies and summarizes the mechanisms underlying the circadian rhythm and the effects of acupuncture characteristics on circadian rhythm. It also describes the neurochemical mechanisms associated with acupuncture treatment of CRSWDs from the standpoint of the central and peripheral biological clocks. Finally, the shortcomings of earlier research on CRSWDs are examined, and contradictory findings about acupuncture are reviewed, and potential further study directions are suggested.

## 2 The mechanism of circadian rhythms

The biological clock, which comprises a central and peripheral biological clock, autonomously regulates the organism’s periodic oscillation of the cycle for approximately 24 h. Circadian clock genes and the proteins they express constitute the core of the circadian rhythm, which is an autonomous transcription-translation oscillation circuit. External stimuli, such as light, temperature, and food, can advance or delay circadian rhythms by aligning with the phase of the endogenous rhythmic oscillation system. These stimuli are referred to as zeitgebers, and the process is known as entrainment.

Light, which is the most important zeitgeber, projects external light–dark information onto the retina. Intrinsically photosensitive retinal ganglion cells then transmit this photic information via the retinohypothalamic tract (RHT) to the suprachiasmatic nucleus (SCN), which acts as the biorhythm’s pacemaker, and subsequently coordinates the other central and peripheral biological clocks through neuro-humoral signals.

**Figure 1:**
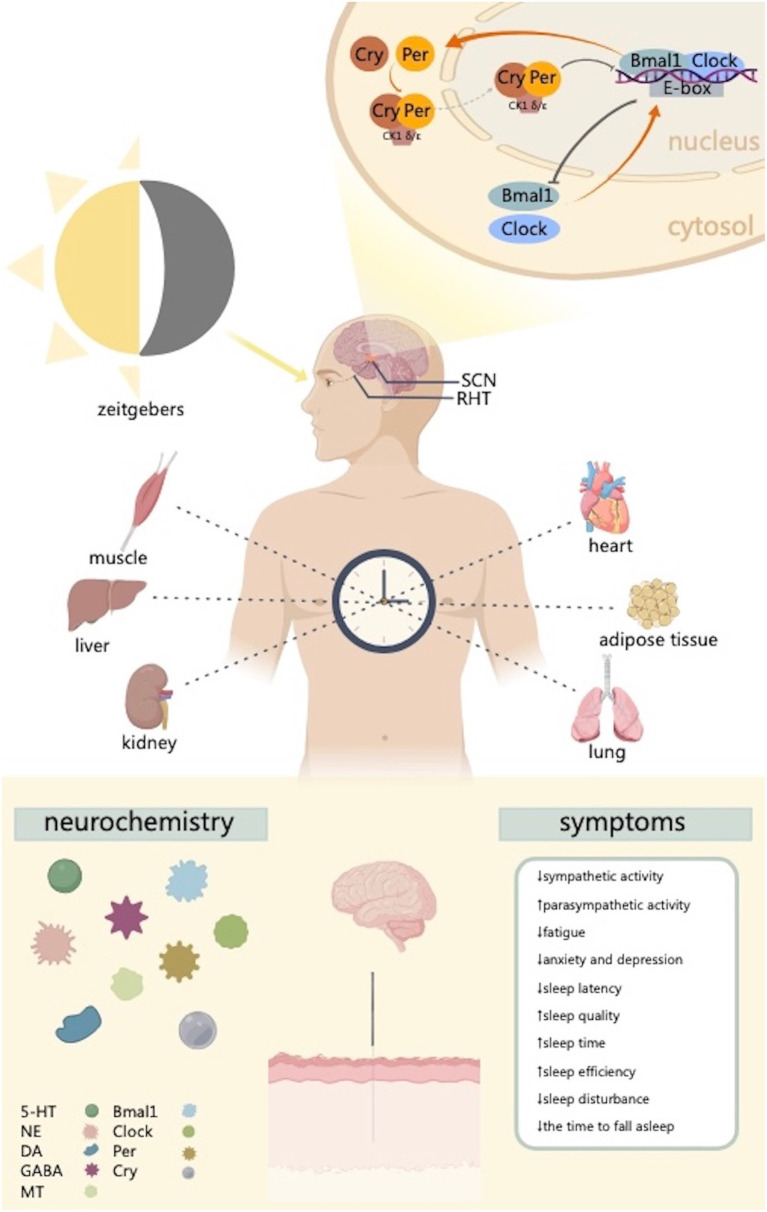
This figure illustrates the way the regulation of circadian clock genes in the SCN and peripheral tissues provides the basis for the formation of circadian rhythms. The biological clock is made up of several clock genes and pertaining proteins. Molecular transcription and translation accurately regulate these genes’ expression, resulting in oscillations of circadian rhythms. This process mainly involves heterodimeric transcriptional activator complex Clock-Bmal1 and the restrictive complex CK1 δ/ε-Cry-Per. Acupuncture can regulate circadian clock genes and improve sleep related symptoms. CK1 δ/ε, casein kinase 1 delta/epsilon; SCN, suprachiasmatic nucleus; RHT, retinohypothalamic tract; 5-HT, serotonin; NE, norepinephrine; DA, dopamine; GABA, γ-aminobutyric acid; MT, melatonin. Created with MedPeer (www.medpeer.cn).

The SCN, the key brain region in the regulation of circadian rhythms, is composed of a cell-autonomous transcription-translation negative feedback loop along with Clock, Bmal1, Per (Per1 and Per2), Cry (Cry1 and Cry2), other circadian clock genes, and their transcription protein products (Takahashi et al., 2008). By phosphorylating proteins implicated in the biological clock, such as Per, Cry, and Bmal1, and controlling protein activity and nuclear localization, casein kinase 1 delta/epsilon (CK1 δ/ε) plays a significant role in the transcriptional-translational feedback loop of the biological clock (Laothamatas et al., 2023). The heterodimer complexes formed by Bmal1 and Clock interact with the promoter region of the E-box to activate the transcription of Per and Cry into Per1, Per2, Cry1, and Cry2 proteins. Consequently, Per and Cry proteins, in turn, form Per-Cry heterodimers and translocate to the nucleus, where they can inhibit the activity of Clock-Bmal1 heterodimer complexes, thereby suppressing gene transcription. This inhibition progressively subsides as the Per-Cry heterodimers decompose, and a new cycle of transcription, approximately 24 h, is re-initiated by Clock-Bmal1 at approximately 24 h (Figure 1; Takahashi et al., 2008; Mohawk et al., 2012; Lande-Diner et al., 2013; Takahashi, 2015).

## 3 Analysis of RCTs and *in vivo* studies on acupuncture for CRSWDs

### 3.1 Search strategy

Relevant randomized controlled trials (RCTs) or *in vivo* studies were included if they met the inclusion criteria of CRSWDs standardized by ICSD-III, and references such as reviews, case reports, and conference papers were excluded. Intervention methods are constrained to simple acupuncture treatment (needles, acupoint selection, manipulations, retention time, and course of treatment are not limited).

**Figure 2:**
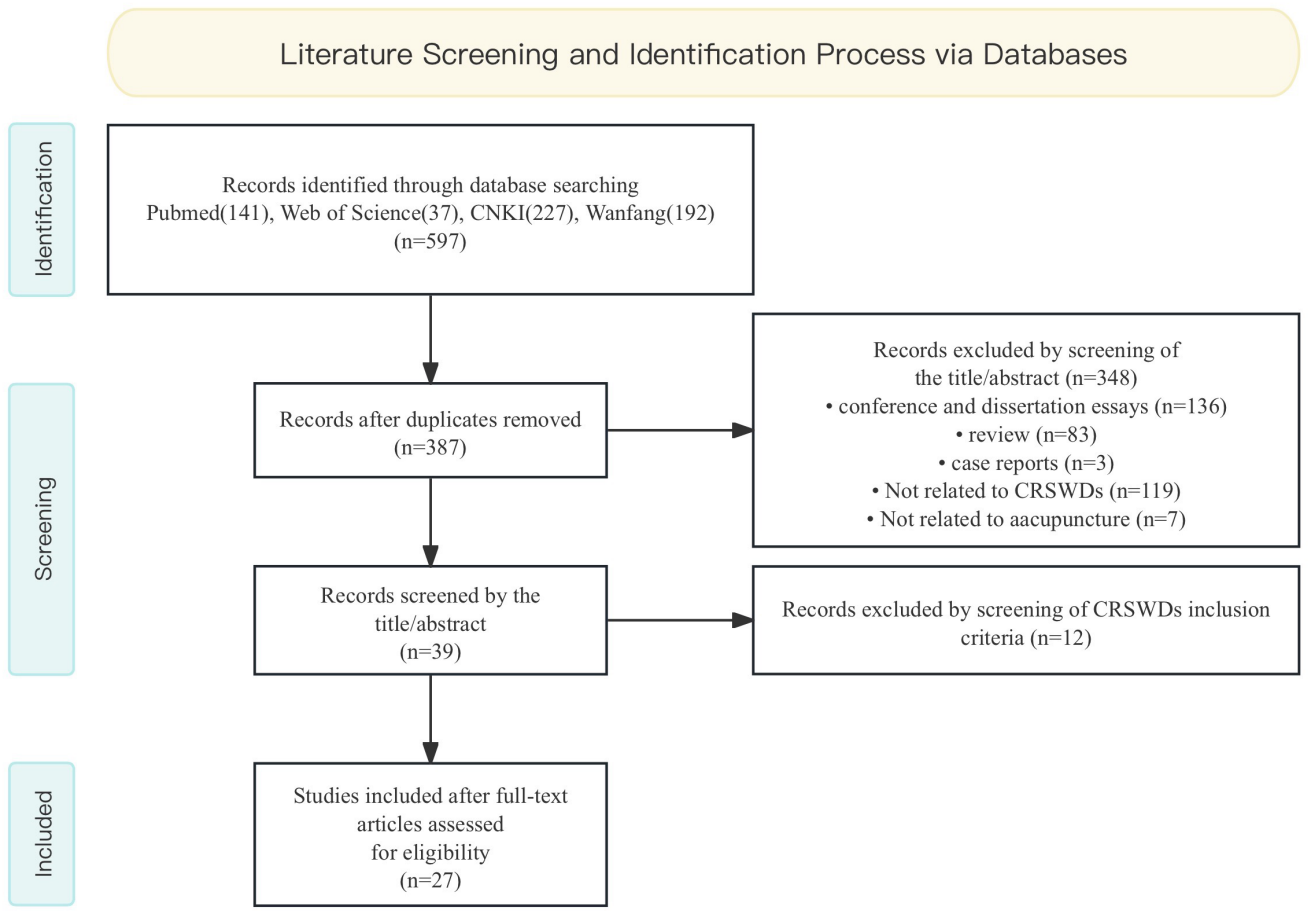
Flow chart of the literature screening process.

We searched Pubmed and Web of Science from database inception through November 2023. For Chinese articles, we searched the China National Knowledge Infrastructure (CNKI) and Wanfang. The keywords used for the search were “circadian rhythm OR circadian rhythm sleep–wake disorders OR CRSWDs” AND “acupuncture” in each database language. The search strategy was adjusted for each database. The flow chart of study searching and screening is shown in Figure 2.

### 3.2 Evidence on the effectiveness of acupuncture in the treatment of CRSWDs

Tables 1, 2 summarize the evidence supporting the effectiveness of acupuncture for treating CRSWDs regarding acupuncture methods, acupoint selection, and physiological mechanisms from *in vivo* and clinical trials.

**Table 1:** Evidence on the effectiveness of acupuncture for treatment of crswds from *in vivo* studies.

Ref.	Model	Model method	Protocol of acupuncture	Major findings			Interpretation
				Insomnia	Other symptoms	Biochemical indicators	
Huang et al. (2023)	adult male SD rats	induced by PCPA	Acupuncture (Ziwuliuzhu Najia method): KI10, LR3, PC7, SP1, LU8, KI2, and LR3 -reinforcing-reducing method -15 min × 7 days -najia group began at 9:00 a.m., nazi group bagan at 11:00 a.m.	↓sleep latency ↓the time to fall asleep ↑sleep time	↓the daily activity ↓jumping and impact movements ↓hyperactivity and mania ↑the sensitivity of responses	↓TNF-α ↓the damage of neurons in the hypothalamic area ↑MT ↑the mRNA expression of Clock and Bmal1	N/A
Lu et al. (2021)	male Balb/c mice	kept in constant DD for 10 days	EA (sparse-dense wave, 2/15 Hz, 0.5 mA): GV1, GV20 -15 min -treated at CT0, CT4, CT8, CT12, CT16, CT20 respectively	phase shift -advanced at CT4 and CT8 -delayed at CT16	N/A	revealed a complete profile of phosphorylation modification in response to EA	EA treatment elicits partial reparation of circadian rhythm exposed to constant darkness.
Guo et al. (2017)	male SD rats	induced by PCPA	acupuncture: BL62 and KI6 -deqi, reinforcing KI6 and reducing BL62 -15 min × 7 days	N/A	↓activity in the rest period	↑the mRNA expression of Per1 and Per2	N/A
Wei et al. (2017)	male SD rats	induced by PCPA	acupuncture: GV20-HT7, GV20-SP6, and GV20-nonacupoint -even reinforcing and reducing -30 min × 7 days	↓sleep latency ↑sleep time	N/A	↑the mRNA expression of Clock and Bmal1 in the area of VLPO and SCN	the effect of acupuncture on GV20-HT7 was better than the other two acupuncture groups.
Liu et al. (2002b)	Wistar adult rats	inverting the L-D rhythm: transforming L/D-12 h into D/L-12 h	acupuncture: parietal sculp acupuncture -at 7 a.m. −10 min	N/A	N/A	↑plasma NE and DA rhythmic phases ↑restoration of brainstem 5-HT and 5-HIAA	the effects on changes in plasma NE and DA levels are not statistically significant.
Cai et al. (2011)	Syrian hamsters	turn on the lights 8 h in advance every 2 days×5 times (10 days in total): to create advanced light–dark cycle	EA (sparse-dense wave, 15 ~ 15 Hz, 1 ~ 3 V): GV1, GV20 −15 min × 3 days -treated at ZT0, ZT4, ZT8, ZT12, ZT16, ZT20 respectively	N/A	N/A	-acupuncture at ZT16 and ZT0, the time of resynchronization was less than the natural recovery group (with no difference in statistics) -acupuncture at ZT4, ZT8 and ZT12 greatly promotes circadian rhythm resynchronization -acupuncture at ZT20, the time of resynchronization in the acupuncture group was longer than that in the natural recovery group (with no difference in statistics)	acupuncture could promote circadian rhythm resynchronization, and the effective of acupuncture on resynchronization was circadian-dependent effects.
Wang et al. (2016)	male SD rats	induced by PCPA	acupuncture: BL62 and KI6 -deqi, reinforcing KI6 and reducing BL62 −20 min × 7 days -between 8 and 10 a.m.	L-D cycle resynchronization of spontaneous activity circadian rhythms	↓daily spontaneous activities	N/A	N/A
Xiu et al. (2021)	C57BL/6 J male rats	perform light stimulation every 2 h × 5 days	EA (sparse-dense wave, 4/15 Hz): BL18 −15 min × 5 days -between 3 and 5 p.m.	N/A	↑spontaneous activities and standing	restore the circadian rhythm of Cry1 mRNA and Cry2 mRNA gene expression in the live ↑contents of Cry1 and Cry2 protein in the liver	N/A
Xu et al. (2023)	male SD rats	induced by PCPA	acupuncture: BL62 and KI6 −20 min × 5 days	EEG and EMG -○REM sleep -↑NREM sleep mesor -↓W mesor	N/A	↑AANAT mRNA expression in PG at ZT16 and ZT20	EEG and ECG suggested a trend toward higher amplitude and a forward shift acrophase, but there was no statistically significant difference.
Xue et al. (2010)	Wistar adult rats	invert the L-D rhythm: transform L/D-12 h into D/L-12 h	EA (sparse-dense wave, 10/20 Hz, 1 ~ 3 V): GV1, GV20 −15 min × 1 day	N/A	inverted-phase HR rhythms return to normal circadian rhythms	N/A	N/A
(Tao et al., 2014b)	SD rats	turn on the lights 8 h in advance every 2 days×5 times (10 days in total): to create advanced light–dark cycle	EA (sparse-dense wave, 15 ~ 20 Hz, 1 ~ 3 V): PC6 and ST36 −15 min × 3 days -treated at ZT4 (11 a.m.)	N/A	N/A	↓MT	N/A
Tao et al. (2014a)	SD rats	turn on the lights 8 h in advance every 2 days×5 times (10 days in total): to create advanced light–dark cycle	EA (sparse-dense wave, 15 ~ 20 Hz, 1 ~ 3 V): PC6 and ST36 −15 min × 3 days -treated at ZT4 (11 a.m.)	N/A	N/A	↑the expression level of PK2 in SCN	N/A
Wang et al. (2023)	C57BL/6 J male rats	alternate light and dark every 2 h	EA (sparse-dense wave, 2/20 Hz, 0.2 mA): BL18 −20 min × 5 days	N/A	N/A	↓expression level of Clock mRNA and Bmal1 mRNA (restoring to normal circadian rhythm)	N/A
Kimura and Hara (2008)	male beagles	alternate light and dark every 2 h	EA (restangular wave, 2 Hz, 5 mV, 250 μsec): GV5 and GV20 −15 min × 1 day	N/A	advancing and activating rhythm of sympathetic nervous activities -↑HR -↓CVRR -↓HF -↓LF:HF ratios	N/A	N/A
Gao et al. (2007)	Kunming white mice	induced by caffeine	acupuncture: EX-HN1 or ST36 -even reinforcing and reducing -without retianing×7 days	↓spontaneous activities during sleep	N/A	↑NOS activity and NO content in the brain	results in EX-HN1 group were all better than those in ST36 group.
Liu et al. (2002a)	Wistar adult rats	inverting the L-D rhythm: transforming L/D-12 h into D/L-12 h	acupuncture: parietal sculp acupuncture -at 7 a.m. −10 min	N/A	N/A	restoration of NE and DA rhythmic phases in the diencephalon -↑NE amplitude -↑DA mesor restoration of 5-HT and 5-HIAA in the diencephalon -↑5-HT amplitude -↑5-HIAA mesor	N/A
Xie et al. (2018)	male SD rats	induced by PCPA	acupuncture: GV20-HT7, GV20-SP6, or GV20-nonacupoint −30 min (rotating every 10 min for 1 min) × 7 days	↓sleep latency ↑sleep time	↑dietary intake ↓water intake feces: restoring to normal color with hardening texture	↑MT level in VLPO ↑expression level of MT1 mRNA and MT2 mRNA in VLPO	results in GV20-HT7 group were all better than those in the other two group.

Symbols: ↓: decreasing; ↑: increasing; ○: no significant statistically difference. SD, Sprague Dawley; PCPA, 4-Chloro-DL-phenylalanin; TNF-α, tumor necrosis factor-α; MT, melatonin; EA, electroacupuncture; CT, circadian time; CT0, 07:00; CT4, 1:00; CT8, 15:00; CT12, 19:00; CT16, 23:00; CT20, 03:00; VLPO, ventrolateral preoptic nucleus; SCN, suprachiasmatic nucleus; L, light; D, dark; NE, norepinephrine; DA, dopamine; 5-HT, serotonin; 5-HIAA, 5-hydroxyindoleacetic acid; REM sleep, rapid eye movement sleep; NREM sleep, none rapid eye movement sleep; W, wake; AANAT, arylalkylamine N-acetyltransferase; PG, pineal body; PK2, prokineticin 2; HR, heart rate; CVRR, coefficient of variation in the R-R intervals; HF, high frequency; LF, low frequency; NOS, nitric oxide synthase; NO, nitric oxide.

**Table 2:** Evidence on the effectiveness of acupuncture for treatment of CRSWDs from clinical studies.

Ref.	Model		Study design			Major findings			Interpretation
		Country	Study design	Protocol of acupuncture	Control group	Insomnia	Other symptoms	Biochemical indicators	
Hwang et al. (2011)	Night-shift nurses	Korea	pilot study: crossover design	Saam acupuncture: GB43, BL66, GB44, and LI4 -manual tonification technique −15 min/session −1 month in total	sham acupuncture	N/A	HRV ↑HF activity ↓LF activity ↓LF:HF ratios	↓sympathetic activity ↑parasympathetic activity	N/A
Hong et al. (2015)	Patients with CRSWDs	China	RCT	acupuncture: SP6, HT7, and EX-HN1 -deqi, even reinforcing and reducing −30 min × 7 days/course −1 courses	tranquilizing oral decoction	↓SRSS	N/A	N/A	N/A
Song et al. (2022)	Patients with insomnia	China	RCT	EA (sparse-dense wave, 5/25 Hz): HT7, SP6, and HT7-SP6 -deqi, even reinforcing and reducing −30 min × 5 days/course −2 courses	those three groups serve as control groups for each other	↓PSQI -↑sleep quality -↑sleep time -↑sleep efficiency -↓sleep disturbance -↓the time to fall asleep ↓ESS	↓daytime fatigue and sleepiness	↓ACTH ↓CS ↑MT	the compatibility of HT7 and SP6 has a synergism effect on the daytime fatigue and sleepiness of insomnia patients, and SP6 has a better effect than HT7.
Yang et al. (2023)	Mobile phone addiction patients with sleep disorders	China	RCT	acupuncture: GV20, GV24, GV29, EX-HN22, Li4, PC6, HT7, and SP6 -deqi, rotating, or lifting manipulation −30 min × 7 days	healthy volunteers with no treatment	↓PSQI -↓sleep quality scores -↓sleep latency scores -↓daytime dysfunction scores	↓MPATS ↓withdrawal symptoms	salivary metabolites in dysrhythmic were restored (including melatonin, thymidine, 2-deoxyuridine, et al.)	N/A
Mou et al. (2023)	Patients with DSWPD	China	RCT	acupuncture: BL62, KI6, LI4, LR3, ST36, and SP6 -deqi, even reinforcing and reducing −20 min × 3 days/week in the 1st to 4th weeks; 20 min × 2 days/week in the 5th to 8th weeks	sham acupuncture (without penetrating through skin)	↓PSQI -↓sleep quality score -↓sleep time score -↓sleep efficiency score -↓sleep disturbance score -↓the time to fall asleep score	↓HAMA ↓HAMA-17	↑plasma MT	N/A
Spence et al. (2004)	adults with insomnia and anxiety	Toronto	an open pre-post clinical trial study	acupuncture -deqi, even reinforcing and reducing −60 min × 2 days/week −5 weeks in total	N/A	PSG -↓sleep onset latency -↑sleep efficiency -↑the total sleep time -↓the arousal index -○the percentage of REM sleep and REM latency ↓the Alpha rating ↓SSS	tests of mood and cognitive efficiency -○the Toronto Alexithymia Scale -↓SSS -↓Fatigue Scale anxiety -↓the State–Trait Anxiety Inventory depression -↓CESD alertness -↓ZOGIM-A ○The composite fatigue scores (comfatigue) -FSS -TWHFQ -ESS -the Fatigue Scale -the FaST Adjective Checklist	the level of aMT6s ↑at night ↓during the morning and afternoon	SSS and fatigue scale scores before sleep did not show any significant differences, but scores indicated a significant improvement after 5 weeks of acupuncture; alertness decreased following acupuncture may imply the substitution of one type of alertness for another rather than representing a decrement in cognitive efficiency.
Yu et al. (2023b)	patients with DSWPD	China	RCT: single blind	acupuncture: BL62, KI6, LI4, LR3, ST 36, and SP6 -deqi, even reinforcing and reducing −20 min × 3 days/week in the 1st to 4th weeks; 20 min × 2 days/week in the 5th to 8th weeks −8 weeks in total	sham acupuncture (without penetrating through skin)	indexes of objective sleep collected by ACT -↑sleep time -↑sleep efficiency -↓the number of awakenings and the wake time after falling asleep indexes/scores of subjective sleep -↑MEQ -↓ISI -↓ESS	↓FSS	↓plasma CORT level	the effect of acupuncture was still obvious in the follow-up of 1, 3 months.
Li and You (2023)	patients with post-stroke CRSWDs	China	RCT	acupuncture (HE’s SanTong acupuncture method): -GV20, GV24, EX-HN1, LI4, PC6, and SP6: deqi, even reinforcing and reducing, 30 min × 3 days/week -BL15, BL13, BL20, BL18, BL23, and BL17: fire needling, ×2 days/week -ear apex bloodletting×3 days/week	oral administration of Estazolam tablets	↓PSQI PSG -↑sleep time -↑REM latency -↑SWS -↓arousal times -↑sleep efficiency	↓HAMA	N/A	N/A
Li et al. (2022)	patients with ischemic stroke CRSWDs	China	RCT	acupnucture (Heart-Brain co-therapy acupunctur method): GV20, EX-HN1, EX-HN3, HT7, PC6, and Anmian acupoint -deqi, even reinforcing and reducing, −30 min × 5 days/week −3 weeks in total	oral administration of Estazolam tablets	↑AIS ↑ESS	N/A	N/A	N/A
Wu et al. (2009)	night-shift male workers	China	RCT: double-blind	laser acupuncture (low-energy laser device, in a treatment dose of 9.7 J/cm2): PC6 -between 7 and 10 p.m. -10 min per time	sham laser acupuncture (power off)	N/A	the autonomic nervous function -○blood pressure -↓heart rate -↓HRV ·↑HF activity ·↓LF activity ·↓LF:HF ratios	↓symp athetic hyperactivities	-heart rate showed no significant difference in treatment group after the 30-min rest period. -the effect of laser acupuncture at PC6 was maintained for at least 30 min after treatment.

Symbols: ↓: decreasing; ↑: increasing; ○: no significant statistically difference. HRV, heart rate variability; HF, high-frequency; LF, low-frequency; SRSS, self-rating scale of sleep; EA, electroacupuncture; PSQI, particularly sleep quality and sleep latency; ESS, the Epworth Sleepiness Scale; ACTH, serum adrenocorticotrophic hormone; CS, cortisol; MT, melatonin; MPATS, MPA tendency scale; Hamilton anxiety scale, HAMA; HRV, Heart Rate Variability; PSG, polysomnography; REM, rapid eye movement; the Alpha rating: an evaluative index of sleep quality; SSS, the Stanford Sleepiness Scale; CESD, The Center for Epidemiological Studies Depression Scale; FSS, the Fatigue Severity Scale; TWHFQ, the Toronto Western Hospital Fatigue Questionnaire; aMT6s, 6-sulfatoxymelatonin; DSWPD, delayed sleep–wake phase disorder; MEQ, morningness-eveningness questionnaire; ISI, Insomnia Severity Index; CORT, cortisol; SWS, slow wave sleep; AIS, Asymptomatic Sleep Disturbance Scale; ZT, zeitgeber time; CESD, The Center for Epidemiological Studies Depression Scale.

## 4 Acupoint selection in the treatment of CRSWDs

Syndrome differentiation and treatment are among the most important components of the basic theory of TCM. Even though patients’ symptoms may be similar, acupuncturists choose different acupoints in accordance with patients’ individual differences under the guidance of TCM theory. HT7, PC6, and SP6 are often used as the main acupoints for insomnia treatment. The effectiveness of the three acupoints alone or in combination in the treatment of CRSWDs has been verified.

Wu et al. found that acupuncture at PC6 affects circadian rhythms in night shift workers by modulating the autonomic nervous system, increasing vagal activity and inhibiting of cardiac sympathetic nerves (Wu et al., 2009). In an RCT, acupuncture at HT7 or SP6 led to significant reductions in Pittsburgh Sleep Quality Interview (PSQI) scores, as well as in serum levels of cortisol (CS) and adrenocorticotrophic hormone (ACTH), compared with pre-treatment levels. However, these two acupoints had varying degrees of effects on different aspects of sleep. The HT7 group exhibited improved scores for daytime dysfunction, sleep disturbance, sleep quality, sleep duration, and sleep efficiency compared to those before treatment. The SP6 group showed improved sleep quality, sleep onset time, sleep duration, sleep efficiency, sleep disturbances, and daytime dysfunction after treatment. In addition, this group outperformed the HT7 group in terms of sleep efficacy and quality. This study also found that the ST7-SP6 combination was more advantageous in the synergistic improvement of daytime sleepiness and fatigue in patients with insomnia. The PSQI and Epworth Sleepiness Scale (ESS) scores, as well as the levels of ACTH, CS, and MT were more favorable than those acquired in the ST7 or SP6 groups alone (Song et al., 2022). However, there is no direct evidence of the underlying mechanism of acupuncture with a single acupoint or acupoint combination in CRSWDs. Further research on the mechanisms of acupuncture in treating CRSWDs is needed.

## 5 The characteristics of acupuncture regulating circadian rhythm

Several functions of organisms are characterized by periodic rhythms to adapt to changes in the external environment. According to TCM theory, qi and blood in the human body undergo periodic variation following the four seasons and 24 h daily. Arnold’s biological tide theory states that changes in people’s emotions and behaviors are correlated with the synodic cycle of the moon. Acupuncture has a benign and bi-directional regulatory effect, and its effect largely depends on the functional state of the body. Additionally, the rhythmic changes in the physiological and pathological state are the foundation for the variations in the acupuncture effect. The characteristics of acupuncture in regulating circadian rhythm are mainly reflected in restoring disturbed circadian rhythms and their temporal features.

Studies have shown that acupuncture can regulate disturbed biological circadian rhythms. Hong et al. (2018) found that acupuncture of ST36, SP6, and GV20 on diabetic rats with depression resulted in a significant down-regulation of Per2 gene expression and a decrease in cortisol (CORT) secretion. The amplitude of CORT activity increased and the peak time shifted forward after acupuncture treatment, restoring approximately normal circadian rhythmic changes (Hong et al., 2018). Chen et al. (2017) investigated how electroacupuncture affected the circadian rhythm of morphine-tolerant mice and discovered that this procedure on BL23 increased the amplitude of rhythms in these mice, facilitated the restoration of disrupted amplitude rhythms, and increased the relative expression of Per1 and Per2.

Acupuncture also has a distinct temporal feature. As a non-photogenic zeitgeber, acupuncture has a modulating effect on photogenic zeitgeber. Recently, several studies have explored the intervention effect of different-timing acupuncture by observing spontaneous activity rhythms. This method of selecting different times for acupuncture according to natural and organism rhythms is known as timing acupuncture. Timing acupuncture falls under the category of TCM time medicine, with a rich theoretical foundation. Ancient Chinese have summarized numerous timing acupuncture methods, including the Ziwu Liuzhu Najia method, eight intelligent turtle methods, and eight flight methods, all of which are still widely used to treat various illnesses (Liu and Huang, 2022).

After electroacupuncture was used to treat insomnia rats at circadian time (CT) 0 (07:00), CT4 (11:00), CT8 (15:00), CT12 (19:00), CT16 (23:00), and CT20 (03,00) under dark–dark (DD) conditions, Lu et al. discovered that electroacupuncture could cause a phase shift, which was manifested by large phase advances at CT4 and CT8. At CT16, phase delays were observed; however, there was no difference in the phase data between the other groups. Electroacupuncture has a bidirectional effect in mice with DD-induced insomnia. This suggests that electroacupuncture can partially repair circadian rhythms in patients exposed to constant darkness (Lu et al., 2021). As a non-photic zeitgeber, electroacupuncture modulates photic zeitgebers. Following electroacupuncture at GV20 and GV1, there was a noticeable shift in the spontaneous behavior of the hamster’s circadian rhythm. This shift indicated an advancement in the subjective daytime rhythm phase and a delay in the subjective nocturnal phase. The amplitude induced by electroacupuncture with photic pulse stimulation was significantly lower than that induced by photic pulse stimulation alone, indicating that electroacupuncture attenuates the entrainment of photic stimuli in the spontaneous behavior of hamsters (Cai et al., 2006). In another study, both healthy volunteers and post-stroke patients received acupuncture stimulation at ST36 from 7:00 a.m. to 9:00 a.m. and from 3:00 p.m. to 5:00 p.m. Functional magnetic resonance imaging (fMRI) revealed that brain activation in the two groups was stronger in the morning than that in the afternoon, and that the regions of their activation areas varied between groups. Therefore, the timing may influence the impact of acupuncture in both healthy individuals and post-stroke patients (Gao et al., 2015).

## 6 Acupuncture in the treatment of CRSWDs comorbid disorders

Patients with CRSWDs commonly have fatigue, anxiety, depression, and pain in addition to symptoms of sleep disorders (Wang et al., 2022). Studies suggest that acupuncture is an effective treatment for comorbid insomnia. According to Song et al. electroacupuncture at SP6 and HT7 reduces daytime sleepiness and fatigue in patients with insomnia (Song et al., 2022). When using manual acupuncture at BL62, KI6, LI4, LR3, ST36, and SP6 to treat patients with DSPD, Yu et al. found that the Fatigue Severity Scale (FSS) scores were significantly reduced and daytime fatigue was greatly improved (Yu X. T. et al., 2023). In an RCT on acupuncture for the treatment of insomnia comorbid diseases, acupuncture at HT7 and KI7 not only improved sleep quality and modified sleep structures, but also had obvious therapeutic effects on fatigue, anxiety, and depression. No serious adverse reactions were observed, suggesting that acupuncture is safe and effective (Wang et al., 2021). Acupuncture is also frequently used to treat insomnia in patients with carcinoma pain. Garland et al. found that after 8 weeks of acupuncture treatment in cancer survivors, the patients’ quality of life, pain, sleep, mood, and fatigue improved, with the effects lasting for at least 20 weeks (Garland et al., 2019).

## 7 Acupuncture modulation of the peripheral biological clock and associated genes

Both central and peripheral biological clock rhythms influence the sleep–wake cycle. In most cases, the manifestation of the SCN rhythm occurs 4–12 h before that of the peripheral rhythm except in single cells, in organs, including the heart, stomach, and liver (Zylka et al., 1998; Yamazaki et al., 2000). The SCN’s molecular regulatory mechanisms for peripheral rhythms are similar to those of the periphery, which can independently control rhythms without assistance from the central biological clock and to counteract the effects of central circadian rhythms (Welsh et al., 2004; Dibner et al., 2010). The peripheral circadian clock, which is widely distributed in tissues and organs including the liver, lungs, kidneys, heart, adipose tissue, and skeletal muscle, responds to upstream signals from the SCN. It also participates in the regulation of the circadian rhythm, hormones, sleep, and heart rate in addition to receiving central signals from the SCN (Honma, 2018; Chowdhury et al., 2019; Patke et al., 2020) by regulating non-optical zeitgebers, such as stress, exercise, and food intake (Ruiter et al., 2003; Tahara and Shibata, 2018).

Studies have shown that acupuncture can stimulate the peripheral biological clock by affecting clock genes and their associated protein expressions. Wang et al. discovered that the livers of rats exhibited chronological changes in the Clock and Bmal1, improving the rats’ circadian rhythm. These changes may have their origins in the downward adjustment of the Clock and Bmal1 genes and their pertaining protein expression (Wang et al., 2023). Researchers have found that acupuncture could reduce the levels of Cry1 and Cry2 and restore the circadian rhythm of Cry1 and Cry2 mRNA gene expression in the livers of mice (Xiu et al., 2021). It has also been demonstrated that acupuncture can drastically lower the expression of the Bmal1, Clock, and Per2 and their pertaining proteins in the cardiomuscular tissue of spontaneous hypertensive rats, as well as a decreasing trend in the levels of Bmal1 and Clock genes in cartilage tissue (Shu et al., 2021; Tan et al., 2022).

## 8 Acupuncture modulation of the central biological clock and pertaining genes

The biological clock controls the body’s circadian rhythm oscillation system, which consists of the central biological clock and the peripheral biological clock. The SCN, the pacemaker of the biological clock, along with the Clock, Bmal1, Per, Cry, and their transcription proteins, make up the autonomous cellular transcription-translation feedback loop. Acupuncture regulates circadian rhythms by regulating the expression of SCN-associated circadian clock genes and proteins. In addition to reducing sleep latency and lengthening sleep time, a study found that acupuncture for insomniac male Sprague Dawley (SD) rats can also reduce the expression level of tumor necrosis factor-α (TNF-α), thereby alleviating inflammatory responses and decreasing structural damage in the hypothalamus (Wei et al., 2017; Huang et al., 2023). Additionally, it can increase the expression levels of Per1 and Per2 mRNA, as well as Clock and Bmal1 mRNA (Guo et al., 2017). In another study investigating the effects of electroacupuncture on circadian rhythms and the expression of clock genes Per1 and Per2 in morphine-tolerant mice, electroacupuncture induced a restoration of aberrant amplitude rhythms to normal levels and upregulated the relative expression of the Per1 and Per2 genes (Chen et al., 2017). It can be inferred that multiple post-translational regulatory processes, including phosphorylation, must be involved in sustaining the SCN cellular transcription-translation feedback loop. Lu et al. also found that under continuous darkness, the electroacupuncture-induced circadian rhythm phase shift had a synchronous effect on mouse SCN phosphorylation (Lu et al., 2021).

## 9 Neurochemical mechanisms of acupuncture for CRSWDs

### 9.1 Monoamines neurotransmitters

#### 9.1.1 Serotonin

Serotonin (5-HT) is primarily derived from the dorsal raphe nucleus (DRN) in the central nervous system (CNS). 5-HT transmits neural signals by binding to 5-HT receptors, which are composed of seven subtypes: 5-HT1–5-HT7. In addition to 5-HT, a variety of neurotransmitters and neuropeptides are present in the DRN, including γ-aminobutyric acid (GABA), dopamine (DA), norepinephrine (NE), glutamic acid, nitric oxide synthase, and others (Monti, 2010). The sleep–wake cycle regulated by 5-HT depends on its interactions with multiple neurotransmitter systems, which lead to the sleep–wake regulation mechanism of the 5-HT complex. 5-HT has been shown to have different effects on sleep and wakefulness in different brain regions.

Notably, 5-HT is considered to be an excitatory neurotransmitter with pro-arousal effects. In rats with insomnia, subcutaneous injection of 5-HT receptor agonists can improve waking and suppress slow-wave-sleep (SWS) and rapid eye movement (REM) sleep (Monti et al., 1995). The optogenetic activation of DRN 5-HT neurons in mice leads to a rapid transition from non-REM (NREM) sleep to active wakefulness (Moriya et al., 2021). REM sleep is increased in 5-HT1A and 5-HT1B receptor-knockout mice (Boutrel et al., 1999, 2002). In contrast, 5-HT is believed to act as a sleep-promoting agent. One study has shown that rats develop insomnia immediately after microinjection of parachlorophenylalanine (PCPA) into the DRN; however, microinjection of 5-hydroxytryptophan (5-HTP) into the bilateral basolateral amygdala (BLA) on the sixth day can reverse the effect of PCPA (Gao et al., 2002). The bidirectional effect of 5-HT on the sleep–wake cycle may depend on the degree to which the serotonergic system is activated and the timing of activation (Monti, 2011). Consequently, it has been hypothesized that 5-HT is a switch that manages the alternation of sleep–wake phases by coupling the sleep–wake center with the SCN, maintaining the continuity of sleep and wake time, and thus contributing to sleep–wake circadian rhythm regulation (Nakamaru-Ogiso et al., 2012).

The effects of acupuncture on 5-HT levels have been extensively studied in patients with CRSWDs. Acupuncture increases 5-HT levels while lowering the concentration of urinary 5-hydroxyindoleacetic acid (5-HIAA) (Beer et al., 2010; Feng et al., 2023). Furthermore, electroacupuncture can accelerate the synthesis and release of 5-HT and NE via the dorsal lateral cord downstream inhibitory pathway, thereby moderating depressive feelings, pain, and sleep circadian rhythm (Han, 1986). However, both RCT and *in vivo* studies have shown that acupuncture can reduce 5-HT levels (Hong et al., 2020; Liu et al., 2021). Therefore, the mechanism of action of acupuncture on 5-HT in patients with CRSWDs requires further study.

#### 9.1.2 Norepinephrine

The primary area for NE generation and release is the locus coeruleus (LC), and the LC-NE system has long been suggested to play a role in activating and maintaining an alert arousal state. It is generally believed that LC activity is significantly reduced during sleep and that NE levels reach their highest levels during wakefulness. NE release gradually decreases from wakefulness to REM sleep (Shouse et al., 2000; Lena et al., 2005). Through B and A1 receptors, NE in the CNS stimulates wakefulness, but inhibiting NE release in the CNS results in profound sedation (Berridge et al., 2012). However, the regulation of the circadian sleep rhythm by NE is not only reflected at its absolute level; research has revealed that the NE oscillation amplitude can regulate sleep microstructure. The long-term decline in NE promotes a spindle-enriched intermediate state and REM sleep, whereas the short-term decline in NE maintains NREM sleep and activates micro-arousals (Kjaerby et al., 2022).

Acupuncture reduced NE levels and improved sleep quality in PCPA-treated rats (Hong et al., 2020). Studies have demonstrated that electroacupuncture at PC6 can reduce increased cardiac sympathetic nervous activity and decrease cardiac vagal nervous activity caused by immobilization stress, which is a known cause of insomnia. The elevated levels of plasma NE and epinephrine released by the sympathoadrenal-medullary axis are shown to be simultaneously decreased by electroacupuncture (Ye et al., 2023). Several trials have shown that acupuncture increases NE levels (Li et al., 2012). In one systematic review, 6 out of the 12 studies included showed an increase in LC activity following acupuncture stimulation. The inconsistencies in the above results may be related to acupuncture manipulation, stimulation frequency, and other study design factors (Lee and Kim, 2017).

#### 9.1.3 Dopamine

DA, a precursor of NE, is an essential neurotransmitter in the ascending activating system, and is mostly found in the ventral tegmental area (VTA) and substantia nigra pars compacta (SNC). DA was previously thought to be an excitatory neurotransmitter that displayed more firing activity and higher levels during arousal and REM sleep, whereas its levels were much lower during NREM sleep (Dahan et al., 2007; Eban-Rothschild et al., 2016). However, research has revealed that DA interferes with sleep–wakefulness regulation in a bidirectional manner. Arousal is induced by high doses of D2 agonists, whereas sleep is induced at low doses (Monti et al., 1988). A study in rats showed that damage to the SNC-DA afferents projecting into the dorsal striatum via the nigrostriatal pathway activates wakefulness and induces sleep–wake fragments. As a result, it is possible that the nigrostriatal dopaminergic pathway can enhance sleep (Qiu et al., 2016), and that DA may play a unique regional, pathway-specific, or dose-dependent role in the regulation of the sleep–wake cycle.

A previous study has shown that sleep deprivation considerably reduces the amount of DA in the hippocampus. Electroacupuncture at GV20 and ST36 can partially reverse the effects of dopamine reduction, which can help alleviate difficulties in learning and memorizing in sleep-deprived individuals (Chen et al., 2020). Electroacupuncture can significantly increase DA and lower TNF-α levels, so as to alleviate the immunosuppressive response and inflammatory damage of intestinal tissues and organs (Li et al., 2021). In cage-changed rats, electroacupuncture is capable of improving sleep quality by increasing the levels of DA and DA receptors in the hypothalamic–pituitary–adrenal (HPA) axis (Xie et al., 2021).

### 9.2 Amino acid neurotransmitters

#### 9.2.1 γ-Aminobutyric acid

GABA is a distinct neurotransmitter of the ascending inhibitory system, which is widely distributed in the CNS. GABAergic neurons receive information from multiple nerve nuclei, creating synaptic connections in various neural structures that are crucial for promoting food intake, consolidating memory, and regulating sleep–wake cycles (Suyama and Yada, 2018). The ventrolateral preoptic area (VLPO) of the hypothalamus is an important sleep-regulating center containing a large number of GABAergic neurons. Microinjections of L-Glu (an excitatory neurotransmitter) into the VLPO at night is shown to significantly prolong the non-REM sleep time; however, this effect is shown to be diminished after selective blockade of GABA receptors (Cheng et al., 2020). Low GABA levels are common in insomnia patients (Luppi et al., 2013). Clinical medicines that are used to treat insomnia, such as BZDs, inhibit the transmission of the NE in sympathetic nerves by enhancing GABA activity in the brain (Mitchell and Weinshenker, 2010).

Auricular point acupuncture has long-term efficacy in treating patients with primary insomnia by increasing their GABA and Glu levels (Chen et al., 2022). Additionally, anxiety induced by insomnia can be reduced by manual acupuncture or electroacupuncture, causing an increase in GABA levels in the hypothalamus of PCPA rats (Zhang et al., 2023).

### 9.3 Melatonin

MT is an endogenous hormone that is synthesized and released by the pineal gland and plays a key role in the regulation of sleep circadian rhythms. The suprachiasmatic nucleus in front of the hypothalamus receives nerve impulses originating from the retinal neurons stimulated by changing light signals. The body then actively manages the pineal gland to rhythmically secrete MT (Socaciu et al., 2020). It is well known that the pattern of melatonin secretion over a 24-h period serves as a synchronizer of circadian activity in humans. MT engages in the sleep–wake cycle through two pathways: first, activating MT1 and MT2 receptors to govern the sleep circadian rhythm (Liu et al., 2016), and second, regulating the concentration of GABA through circadian rhythmic secreted changes (Yu Q. et al., 2023). Additionally, MT has been verified to participate in the rhythmic expression of certain circadian clock genes. For example, MT treatment can lower the methylation level of the circadian clock gene Per1 to promote its rhythmic expression (Tsukamoto-Yamauchi et al., 2015), and can also increase Bmal1 expression via various signaling pathways (Sanchez et al., 2018).

Through raising MT levels and lowering TNF-α expression in hypothalamic SCN cells, acupuncture can alleviate the pathological products and inflammatory responses of chronic insomnia (Huang et al., 2023). Acupuncture has a particularly good safety profile and has been shown in a trial to alleviate insomnia in pregnant women by elevating melatonin levels without any significant adverse events (Foroughinia et al., 2020). Another clinical trial found significant improvements in sleep onset latency, total sleep length, and sleep efficiency measured by polysomnography (PSG) after 5 weeks of acupuncture in anxious patients. A considerable increase in endogenous nocturnal MT secretion was also detected (Spence et al., 2004).

## 10 Research limitations and further directions on acupuncture for CRSWDs

As previously mentioned, acupuncture can alleviate symptoms and regulate disturbed circadian rhythms in patients with CRSWDs. However, some studies have proposed that acupuncture for insomnia has insignificant differences in effectiveness compared to sham/placebo acupuncture, and no obvious long-term effects have been observed. Wang et al. found that acupuncture at HT7 and KI7 could alleviate the symptoms of insomnia comorbid conditions (daytime sleepiness, fatigue, anxiety, and depression). Moreover, statistically significant reductions were observed in PSQI and Insomnia Severity Index (ISI) scores within or between the groups (acupuncture and sham acupuncture). Although the PSQI and ISI scores remained lower at follow-up than at baseline, no differences were noted between groups (Wang et al., 2021). Yeung et al. found no significant difference in the ISI score or other outcome measures between patients with primary insomnia in the electroacupuncture and placebo acupuncture groups (Yeung et al., 2009). The differences in needles, acupoints, manipulations, timing, and study participants may cause the inconsistency between these findings and those of other studies. In addition, selecting rating scales for outcome indicators is influenced by the patient’s subjective awareness. Therefore, the absence of apparent distinction between the acupuncture and sham/placebo acupuncture groups may be attributed to the role of acupressure in these studies. Although sham/placebo acupuncture does not penetrate the skin, its mechanism of action is similar to that of acupuncture. Acupressure and acupuncture can modulate the visceral organs and meridians through the physical stimulation of acupoints, thereby treating diseases by harmonizing yin and yang.

Choosing an adequate method to evaluate the benefit of acupuncture for treating CRSWDs is challenging. RCTs conducted on individuals with CRSWDs are more challenging than those performed on mice because they require extended closure times for complete observations of the circadian rhythm. In addition, since clinical research invariably involves the strong timing impacts of zeitgebers (e.g., light, food, and activity), acupuncture’s clinical effectiveness may be lower than that attained in a laboratory setting or a free-run condition (Hou et al., 2021). Currently, objective biochemical indicators, such as MT and its metabolites, and circadian clock gene mRNA expression are widely used to observe the regulation of circadian rhythms at the molecular level. In addition, subjective evaluation scales, such as the PSQI, ISI, and ESS, are used to assess patients’ sleep quality. However, these results significantly vary depending on the patient’s consciousness and susceptibility to the placebo effect. The objective evaluation of sleep quality is one of the most important approaches for increasing the effectiveness of acupuncture in CRSWDs treatment. Techniques, such as PSG and actigraphy, can record objective electronic sleep data. PSG, the gold standard for measuring sleep, collects electroencephalogram (EEG), electrocardiogram (ECG), electromyography (EMG), and other parameters, along with heart rate, breathing, and blood oxygen saturation. It also records other physiological indicators to objectively assess sleep and its stages, including REM and NREM sleep (Reite et al., 1995). Standard PSG monitoring is not widely used to evaluate sleep in patients with insomnia since it must be completed in a laboratory, which usually interferes with a patient’s regular sleep (Littner et al., 2003). Alternatively, actigraphy tracks limb motions and automatically collects sleep characteristics, such as sleep time, sleep efficiency, and wake time (Lichstein et al., 2006). ICSD-III, as edited by the American Academy of Sleep Medicine (AASM), recommends actigraphy rather than ESG when evaluating the multi-day sleep–wake patterns of adults and children (Sateia, 2014).

There is still a lack of RCTs despite the numerous recent studies on the regulation of circadian rhythms by acupuncture. Additionally, the current absence of national and international criteria on CRSWDs poses a challenge since participant inclusion in RCTs is unstandardized, and CRSWDs are frequently confused with other forms of insomnia. Most RCTs have focused on discussing symptoms such as circadian rhythm recovery. However, the studies on the acupuncture mechanism have mostly been limited to animal experiments in laboratory settings, implying that the results cannot be widely applied given the differences between polyphasic sleep in rodents and humans. Advanced techniques, including fMRI, are progressively employed as one of the observational indices for acupuncture treatment of CRSWDs (Gao et al., 2015; Ning et al., 2020). Although the findings point to the responsible brain region, the precise neurochemical process remains unknown. Therefore, more clinical mechanism research should be conducted in the future.

Due to the temporal feature of acupuncture, the timing of when to select acupuncture remains a constant source of concern. In *in vivo* studies, the six-time points of CT0, CT4, CT8, CT12, CT16, and CT20 are frequently used as acupuncture intervention timing, and several studies have shown that the different time points can delay, advance, or synchronize circadian rhythms. However, clinical studies are hampered because the acupuncture time of RCTs is restricted to daytime, hindering a thorough exploration of its temporal features and creating obstacles for clinical research. To better understand the ideal timing of acupuncture for various diseases and to increase its clinical efficacy, researchers shall keep investigating the temporal feature of acupuncture.

Furthermore, current studies have mostly focused on the circadian regulation of the central biological clock by acupuncture, with insufficient attention dedicated to investigating the rhythmic processes of the peripheral biological clock. Therefore, considering the significance of the peripheral biological clock, the potential of acupuncture as a treatment for CRSWDs will be greatly bolstered by discovering the mechanisms or pathways of the peripheral biological clock.

## 11 Conclusion

CRSWDs are a unique category of sleep disorders with a complex mechanism. It is evident that acupuncture, which is free of harmful side effects, can alleviate CRSWDs symptoms. Under the regulation of the central and peripheral biological clock, acupuncture, as a nonphotic zeitgeber, plays an important role in restoring circadian rhythms by modulating SCN, clock genes, and their protein products, as well as neurotransmitters.

Establishing a standardized diagnosis and acupuncture treatment protocol is necessary, including clear instructions on the needles, acupoint selection, manipulations, parameters, retaining time, and courses. Furthermore, the majority of the studies in this review are basic research. Conducting more relevant clinical research, deepening the understanding of the intrinsic mechanisms enables us to further assess the effectiveness and safety of acupuncture for CRSWDs.

## Author contributions

JW: Writing – original draft. ZZ: Writing – review & editing.

## Glossary

CRSWDs: Circadian rhythm sleep–wake disorders
TCM: Traditional Chinese medicine
PSQI: Pittsburgh Sleep Quality Interview
CS: Cortisol
ACTH: Adrenocorticotrophic hormone
D-D: Dark–dark
DSPD: Delayed sleep phase disorder
ASPD: Advanced sleep phase disorder
FSS: Fatigue Severity Scale
ISI: Insomnia Severity Index
BZDs: Benzodiazepines
MT: Melatonin
PSG: Polysomnography
EEG: Electroencephalogram
ECG: Electrocardiogram
EMG: Electromyography
REM: Rapid eye movement
NREM: Non rapid eye movement
AASM: American Academy of Sleep Medicine
5-HT: Serotonin
DRN: Dorsal raphe nucleus
GABA: γ-aminobutyric acid
DA: Dopamine
NE: Norepinephrine
CNS: Central nervous system
PCPA: Parachlorophenylalanine
5-HTP: 5-hydroxytryptophan
BLA: Basolateral amygdala
5-HIAA: 5-hydroxyindoleacetic acid
HPA: Hypothalamic–pituitary–adrenal
LC: Locus coeruleus
VTA: Ventral tegmental area
SNC: Substantia nigra pars compacta
CT: Circadian time
CORT: Cortisol
RHT: Retinohypothalamic tract
SCN: Suprachiasmatic nucleus
RCT: Randomized controlled trial
TNF-α: Tumor necrosis factor-α
fMRI: Functional magnetic resonance imaging
SHR: Spontaneous hypertensive rats
VLPO: Ventrolateral preoptic nucleus
SWS: Slow wave sleep
ICSD: International Classification of Sleep Disorders
ESS: The Epworth Sleepiness Scale
CK1 δ/ε: Casein kinase 1 delta/epsilon

## References

[ref1] Atkin T. Comai S. Gobbi G. (2018). Drugs for insomnia beyond benzodiazepines: pharmacology, clinical applications, and discovery. Pharmacol. Rev. 70, 197–245. doi: 10.1124/pr.117.014381, PMID: 29487083

[ref2] Barion A. Zee P. C. (2007). A clinical approach to circadian rhythm sleep disorders. Sleep Med. 8, 566–577. doi: 10.1016/j.sleep.2006.11.017, PMID: 17395535 PMC2679862

[ref3] Beer T. M. Benavides M. Emmons S. L. Hayes M. Liu G. Garzotto M.. (2010). Acupuncture for hot flashes in patients with prostate cancer. Urology 76, 1182–1188. doi: 10.1016/j.urology.2010.03.033, PMID: 20494414 PMC2928879

[ref4] Berridge C. W. Schmeichel B. E. Espana R. A. (2012). Noradrenergic modulation of wakefulness/arousal. Sleep Med. Rev. 16, 187–197. doi: 10.1016/j.smrv.2011.12.003, PMID: 22296742 PMC3278579

[ref5] Boutrel B. Franc B. Hen R. Hamon M. Adrien J. (1999). Key role of 5-HT1B receptors in the regulation of paradoxical sleep as evidenced in 5-HT1B knock-out mice. J. Neurosci. 19, 3204–3212. doi: 10.1523/JNEUROSCI.19-08-03204.1999, PMID: 10191333 PMC6782285

[ref6] Boutrel B. Monaca C. Hen R. Hamon M. Adrien J. (2002). Involvement of 5-HT1A receptors in homeostatic and stress-induced adaptive regulations of paradoxical sleep: studies in 5-HT1A knock-out mice. J. Neurosci. 22, 4686–4692. doi: 10.1523/JNEUROSCI.22-11-04686.2002, PMID: 12040075 PMC6758830

[ref7] Cai D. Song K. Liu X. Zhou Q. Zhao J. Wei J. (2006). Effect of electro-acupuncture on entraining rhythm effective of photic zeitgeber. J. Sichuan Tradit. Chin. Med. 1, 16–18. doi: 10.3969/j.issn.1000-3649.2006.07.009

[ref8] Cai D. J. Wei J. L. Zhao J. L. Xie C. Liu X. G. Zhou Q. Z. (2011). Research on the phase characteristic of acupuncture on the circadian rhythm disturbance due to advanced light/dark cycle. China J. Trad. Chin. Med. Pharm. 26, 691–694. doi: CNKI:SUN:BXYY.0.2011-04-018

[ref9] Chen S. Xiong P. Xue H. (2017). Electro-acupuncture regulate the circadian rhythms and its expression of clock gene period 1 and period 2 on morphine tolerance mice. Lishizhen Med. Mater. Med. Res. 28, 2019–2022. doi: 10.3969/j.issn.1008-0805.2017.08.085

[ref10] Chen D. Zhang Y. Wang C. Wang X. Shi J. Zhang J.. (2020). Modulation of hippocampal dopamine and synapse-related proteins by electroacupuncture improves memory deficit caused by sleep deprivation. Acupunct. Med. 38, 343–351. doi: 10.1177/0964528420902147, PMID: 32370535

[ref11] Chen H. Zhang M. J. Wu J. A. She Y. F. Yuan X. R. Huo Y. X.. (2022). Effect of auricular Acupoint bloodletting plus auricular acupressure on sleep quality and neuroendocrine level in college students with primary insomnia: a randomized controlled trial. Chin. J. Integr. Med. 28, 1096–1104. doi: 10.1007/s11655-022-3581-0, PMID: 36327047

[ref12] Cheng J. Wu F. Zhang M. Ding D. Fan S. Chen G.. (2020). The interaction between the ventrolateral preoptic nucleus and the Tuberomammillary nucleus in regulating the sleep-wakefulness cycle. Front. Neurosci. 14:615854. doi: 10.3389/fnins.2020.615854, PMID: 33381012 PMC7767984

[ref13] Chowdhury D. Wang C. Lu A. P. Zhu H. L. (2019). Understanding quantitative circadian regulations are crucial towards advancing chronotherapy. Cell 8:883. doi: 10.3390/cells8080883, PMID: 31412622 PMC6721722

[ref14] Dahan L. Astier B. Vautrelle N. Urbain N. Kocsis B. Chouvet G. (2007). Prominent burst firing of dopaminergic neurons in the ventral tegmental area during paradoxical sleep. Neuropsychopharmacology 32, 1232–1241. doi: 10.1038/sj.npp.1301251, PMID: 17151599

[ref15] De Crescenzo F. D'alo G. L. Ostinelli E. G. Ciabattini M. Di Franco V. Watanabe N.. (2022). Comparative effects of pharmacological interventions for the acute and long-term management of insomnia disorder in adults: a systematic review and network meta-analysis. Lancet 400, 170–184. doi: 10.1016/S0140-6736(22)00878-9 35843245

[ref16] Dibner C. Schibler U. Albrecht U. (2010). The mammalian circadian timing system: organization and coordination of central and peripheral clocks. Annu. Rev. Physiol. 72, 517–549. doi: 10.1146/annurev-physiol-021909-135821 20148687

[ref17] Eban-Rothschild A. Rothschild G. Giardino W. J. Jones J. R. De Lecea L. (2016). VTA dopaminergic neurons regulate ethologically relevant sleep-wake behaviors. Nat. Neurosci. 19, 1356–1366. doi: 10.1038/nn.4377, PMID: 27595385 PMC5519826

[ref18] Ernst E. (2006). Acupuncture–a critical analysis. J. Intern. Med. 259, 125–137. doi: 10.1111/j.1365-2796.2005.01584.x 16420542

[ref19] Everitt H. Baldwin D. S. Stuart B. Lipinska G. Mayers A. Malizia A. L.. (2018). Antidepressants for insomnia in adults. Cochrane Database Syst. Rev. 5:Cd010753. doi: 10.1002/14651858.CD010753.pub2 29761479 PMC6494576

[ref20] Feng X. X. Huang K. Y. Chen L. Zhou K. (2023). Clinical efficacy of the shallow puncture and more-twirling acupuncture method in migraine treatment and its effects on serum 5-HT and beta-EP levels. Technol. Health Care 31, 533–540. doi: 10.3233/THC-236047, PMID: 37038799 PMC10200134

[ref21] Foroughinia S. Hessami K. Asadi N. Foroughinia L. Hadianfard M. Hajihosseini A.. (2020). Effect of acupuncture on pregnancy-related insomnia and melatonin: a single-blinded, randomized, placebo-controlled trial. Nat. Sci. Sleep 12, 271–278. doi: 10.2147/NSS.S247628, PMID: 32494210 PMC7231755

[ref22] Gao Y. Lin Z. Tao J. Yang S. Chen R. Jiang C.. (2015). Evidence of timing effects on acupuncture: a functional magnetic resonance imaging study. Exp. Ther. Med. 9, 59–64. doi: 10.3892/etm.2014.2056, PMID: 25452777 PMC4247279

[ref23] Gao X. Y. Ma Q. L. Hu B. (2007). Effects of acupuncture at "Sishencong" (EX-HN 1) on physioloqical functions in the sleep disorder model mouse. Zhongguo Zhen Jiu 27, 681–683. doi: 10.3969/j.issn.1005-7072.2001.05.001 PMID: 17926623

[ref24] Gao J. Zhang J. X. Xu T. L. (2002). Modulation of serotonergic projection from dorsal raphe nucleus to basolateral amygdala on sleep-waking cycle of rats. Brain Res. 945, 60–70. doi: 10.1016/S0006-8993(02)02625-2, PMID: 12113952

[ref25] Garland S. N. Xie S. X. Duhamel K. Bao T. Li Q. Barg F. K.. (2019). Acupuncture versus cognitive behavioral therapy for insomnia in Cancer survivors: a randomized clinical trial. J. Natl. Cancer Inst. 111, 1323–1331. doi: 10.1093/jnci/djz050, PMID: 31081899 PMC6910189

[ref26] Guo B. J. Yu S. Y. Shen Z. F. Hu Y. P. (2017). Effect of acupuncture at points in heel vessel for circadian clock genes of period 1 and period 2 mRNAs in the suprachiasmatic nucleus in insomnia rats. Zhen Ci Yan Jiu 42, 507–509. doi: 10.13702/j.1000-0607.2017.06.007, PMID: 29318856

[ref27] Han J. S. (1986). Electroacupuncture: an alternative to antidepressants for treating affective diseases? Int. J. Neurosci. 29, 79–92. doi: 10.3109/00207458608985638, PMID: 3516903

[ref28] Hong J. Chen J. Kan J. Liu M. Yang D. (2020). Effects of acupuncture treatment in reducing sleep disorder and gut microbiota alterations in PCPA-induced insomnia mice. Evid. Based Complement. Alternat. Med. 2020, 1–14. doi: 10.1155/2020/3626120 PMC7647758 33178314

[ref29] Hong X. J. Wang B. L. Huang J. X. Guo H. (2018). Effects of acupuncture on circadian rhythm of CORT and mPer1/mPer2 genes expression on diabetes mellitus rats with depression. Liaoning J. Tradit. Chin. Med. 45, 483–486. doi: 10.13192/j.issn.1000-1719.2018.03.008

[ref30] Hong J. J. Yu H. J. Zhao C. H. Xu R. G. Zhang T. Zhao H. H.. (2015). Tranquilize acupuncture treatment of circadian rhythm sleep disorders. J. Changchun Univ. Chin. Med. 31, 114–115. doi: 10.13463/j.cnki.cczyy.2015.01.038

[ref31] Honma S. (2018). The mammalian circadian system: a hierarchical multi-oscillator structure for generating circadian rhythm. J. Physiol. Sci. 68, 207–219. doi: 10.1007/s12576-018-0597-5, PMID: 29460036 PMC10717972

[ref32] Hou Y. Liu L. Xiao Y. Chen X. Xiong J. Zhou Y.. (2021). Discussion on the mechanism of acupuncture treatment for circadian rhythm sleep-wake disorders from the perspective of regulating central biological clock. China J. Tradit. Chin. Med. Pharm. 36, 6022–6026.

[ref33] Huang A. Xiao G. Chen Y. Hu Z. Lee P. H. Huang Y.. (2023). Ziwuliuzhu acupuncture modulates clock mRNA, Bmal1 mRNA and melatonin in insomnia rats. J. Acupunct. Meridian Stud. 16, 109–118. doi: 10.51507/j.jams.2023.16.3.109 37381033

[ref34] Hwang D. S. Kim H. K. Seo J. C. Shin I. H. Kim D. H. Kim Y. S. (2011). Sympathomodulatory effects of Saam acupuncture on heart rate variability in night-shift-working nurses. Complement. Ther. Med. 19, S33–S40. doi: 10.1016/j.ctim.2010.11.001, PMID: 21195293

[ref35] Kaptchuk T. J. (2002). Acupuncture: theory, efficacy, and practice. Ann. Intern. Med. 136, 374–383. doi: 10.7326/0003-4819-136-5-200203050-00010, PMID: 11874310

[ref36] Kimura Y. Hara S. (2008). The effect of electro-acupuncture stimulation on rhythm of autonomic nervous system in dogs. J. Vet. Med. Sci. 70, 349–352. doi: 10.1292/jvms.70.349 18460828

[ref37] Kjaerby C. Andersen M. Hauglund N. Untiet V. Dall C. Sigurdsson B.. (2022). Memory-enhancing properties of sleep depend on the oscillatory amplitude of norepinephrine. Nat. Neurosci. 25, 1059–1070. doi: 10.1038/s41593-022-01102-9, PMID: 35798980 PMC9817483

[ref38] Lande-Diner L. Boyault C. Kim J. Y. Weitz C. J. (2013). A positive feedback loop links circadian clock factor CLOCK-BMAL1 to the basic transcriptional machinery. Proc. Natl. Acad. Sci. U. S. A. 110, 16021–16026. doi: 10.1073/pnas.1305980110 24043798 PMC3791755

[ref39] Laothamatas I. Rasmussen E. S. Green C. B. Takahashi J. S. (2023). Metabolic and chemical architecture of the mammalian circadian clock. Cell Chem. Biol. 30, 1033–1052. doi: 10.1016/j.chembiol.2023.08.014, PMID: 37708890 PMC10631358

[ref40] Lee G. Kim W. (2017). The modulatory effect of acupuncture on the activity of locus Coeruleus neuronal cells: a review. Evid. Based Complement. Alternat. Med. 2017, 1–8. doi: 10.1155/2017/9785345 PMC5664286 29234450

[ref41] Lena I. Parrot S. Deschaux O. Muffat-Joly S. Sauvinet V. Renaud B.. (2005). Variations in extracellular levels of dopamine, noradrenaline, glutamate, and aspartate across the sleep–wake cycle in the medial prefrontal cortex and nucleus accumbens of freely moving rats. J. Neurosci. Res. 81, 891–899. doi: 10.1002/jnr.20602, PMID: 16041801

[ref42] Li M. Hu L. Cai R. L. Wu Z. J. Wang K. M. (2012). Effects of electroacupuncture at PC6 and BL15 on nerve electrical activity in spinal dorsal root and norepinephrine and dopamine contents in paraventricular nucleus of hypothalamus in rats with acute myocardial ischemia. Zhong Xi Yi Jie He Xue Bao 10, 874–879. doi: 10.3736/jcim20120807, PMID: 22883403

[ref43] Li C. Y. Jin L. T. Ma Y. Q. Wang J. (2022). Clinical study of heart-brain co-therapy acupuncture in treating sleep rhythm disorders of ischemic stroke. Inf. Tradit. Chin. Med. 39, 60–64. doi: 10.19656/j.cnki.1002-2406.20220112

[ref44] Li Y. Xu G. Hu S. Wu H. Dai Y. Zhang W.. (2021). Electroacupuncture alleviates intestinal inflammation and barrier dysfunction by activating dopamine in a rat model of intestinal ischaemia. Acupunct. Med. 39, 208–216. doi: 10.1177/0964528420922232, PMID: 32517478

[ref45] Li D. You W. (2023). Efficacy observation of HE’s San Tong acupuncture method for post-stroke sleep wake disorders and the impact on sleep quality. Shanghai J. Acupunct. Moxibustion 42, 559–564. doi: 10.13460/j.issn.1005-0957.2023.06.0559

[ref46] Lichstein K. L. Stone K. C. Donaldson J. Nau S. D. Soeffing J. P. Murray D.. (2006). Actigraphy validation with insomnia. Sleep 29, 232–239. doi: 10.1093/sleep/29.2.232 PMID: 16494091

[ref47] Littner M. Hirshkowitz M. Kramer M. Kapen S. Anderson W. M. Bailey D.. (2003). Practice parameters for using polysomnography to evaluate insomnia: an update. Sleep 26, 754–760. doi: 10.1093/sleep/26.6.754, PMID: 14572131

[ref48] Liu J. Clough S. J. Hutchinson A. J. Adamah-Biassi E. B. Popovska-Gorevski M. Dubocovich M. L. (2016). MT1 and MT2 melatonin receptors: a therapeutic perspective. Annu. Rev. Pharmacol. Toxicol. 56, 361–383. doi: 10.1146/annurev-pharmtox-010814-124742, PMID: 26514204 PMC5091650

[ref49] Liu J. Huang F. (2022). Review of TCM time medicine research. China J. Tradit. Chin. Med. Pharm. 37, 5880–5882.

[ref50] Liu Y. X. Song K. Y. Liu X. G. Zhou Q. Z. Zhao J. L. Zeng Z. (2002a). The effect of parietal area scalp acupuncture on the levels of norepinephrine and dopamine in the plasma of rats with inverted activity rhythm. Sichuan Zhongyi 1, 8–9. doi: 10.3969/j.issn.1006-3250.1999.02.017

[ref51] Liu Y. X. Song K. Y. Yu S. G. Liu X. G. Zhou Q. Z. (2002b). The effect of parietal scalp on monoamine neurotransmitters in the brainstem of rats with inverted rhythmic activity. Sichuan Zhongyi 1, 7–9. doi: 10.3969/j.issn.1000-3649.2002.03.004

[ref52] Liu C. Zhao Y. Qin S. Wang X. Jiang Y. Wu W. (2021). Randomized controlled trial of acupuncture for anxiety and depression in patients with chronic insomnia. Ann. Transl. Med. 9:1426. doi: 10.21037/atm-21-3845, PMID: 34733978 PMC8506741

[ref53] Lu X. Zhou M. Liu N. Zhang C. Zhao Z. Cai D. (2021). Synaptic protein phosphorylation networks are associated with Electroacupuncture-induced circadian control in the suprachiasmatic nucleus. Front. Genet. 12:762557. doi: 10.3389/fgene.2021.762557, PMID: 34976011 PMC8717940

[ref54] Luppi P. H. Clement O. Valencia Garcia S. Brischoux F. Fort P. (2013). New aspects in the pathophysiology of rapid eye movement sleep behavior disorder: the potential role of glutamate, gamma-aminobutyric acid, and glycine. Sleep Med. 14, 714–718. doi: 10.1016/j.sleep.2013.02.004, PMID: 23790501

[ref55] Mitchell H. A. Weinshenker D. (2010). Good night and good luck: norepinephrine in sleep pharmacology. Biochem. Pharmacol. 79, 801–809. doi: 10.1016/j.bcp.2009.10.004, PMID: 19833104 PMC2812689

[ref56] Mohawk J. A. Green C. B. Takahashi J. S. (2012). Central and peripheral circadian clocks in mammals. Annu. Rev. Neurosci. 35, 445–462. doi: 10.1146/annurev-neuro-060909-153128, PMID: 22483041 PMC3710582

[ref57] Monti J. M. (2010). The structure of the dorsal raphe nucleus and its relevance to the regulation of sleep and wakefulness. Sleep Med. Rev. 14, 307–317. doi: 10.1016/j.smrv.2009.11.004, PMID: 20153669

[ref58] Monti J. M. (2011). Serotonin control of sleep-wake behavior. Sleep Med. Rev. 15, 269–281. doi: 10.1016/j.smrv.2010.11.003 21459634

[ref59] Monti J. M. Hawkins M. Jantos H. D'angelo L. Fernandez M. (1988). Biphasic effects of dopamine D-2 receptor agonists on sleep and wakefulness in the rat. Psychopharmacology 95, 395–400. doi: 10.1007/BF00181955, PMID: 3137628

[ref60] Monti J. M. Jantos H. Silveira R. Reyes-Parada M. Scorza C. (1995). Sleep and waking in 5,7-DHT-lesioned or (−)-pindolol-pretreated rats after administration of buspirone, ipsapirone, or gepirone. Pharmacol. Biochem. Behav. 52, 305–312. doi: 10.1016/0091-3057(94)00414-E 8577795

[ref61] Moriya R. Kanamaru M. Okuma N. Yoshikawa A. Tanaka K. F. Hokari S.. (2021). Optogenetic activation of DRN 5-HT neurons induced active wakefulness, not quiet wakefulness. Brain Res. Bull. 177, 129–142. doi: 10.1016/j.brainresbull.2021.09.019 34563634

[ref62] Mou Y. Y. Zhao N. Xie C. Bi Y. P. Sun X. Q. Sun W. J.. (2023). Efficacy observation of acupuncture for delayed sleep-wake phase disorder and its effect on sleep, mood, and plasma melatonin level. Shanghai J. Acupunct. Moxibustion 42, 466–471. doi: 10.13460/j.issn.1005-0957.2023.05.0466

[ref63] Nakamaru-Ogiso E. Miyamoto H. Hamada K. Tsukada K. Takai K. (2012). Novel biochemical manipulation of brain serotonin reveals a role of serotonin in the circadian rhythm of sleep-wake cycles. Eur. J. Neurosci. 35, 1762–1770. doi: 10.1111/j.1460-9568.2012.08077.x 22625848

[ref64] Ning Y. Liu X. Yao H. Chen P. Li X. Jia H. (2020). The fMRI study for acupuncture on shift work sleep disorder: study protocol for a randomized controlled neuroimaging trial. Medicine (Baltimore) 99:e22068. doi: 10.1097/MD.0000000000022068, PMID: 32899073 PMC7478636

[ref65] Paine S. J. Fink J. Gander P. H. Warman G. R. (2014). Identifying advanced and delayed sleep phase disorders in the general population: a national survey of New Zealand adults. Chronobiol. Int. 31, 627–636. doi: 10.3109/07420528.2014.885036, PMID: 24548144

[ref66] Patke A. Young M. W. Axelrod S. (2020). Molecular mechanisms and physiological importance of circadian rhythms. Nat. Rev. Mol. Cell Biol. 21, 67–84. doi: 10.1038/s41580-019-0179-2 31768006

[ref67] Qiu M. H. Yao Q. L. Vetrivelan R. Chen M. C. Lu J. (2016). Nigrostriatal Dopamine Acting on Globus Pallidus Regulates Sleep. Cereb. Cortex 26, 1430–1439. doi: 10.1093/cercor/bhu241, PMID: 25316334 PMC4785943

[ref68] Reite M. Buysse D. Reynolds C. Mendelson W. (1995). The use of polysomnography in the evaluation of insomnia. Sleep 18, 58–70. doi: 10.1093/sleep/18.1.58 7761745

[ref69] Ruiter M. La Fleur S. E. Van Heijningen C. Van Der Vliet J. Kalsbeek A. Buijs R. M. (2003). The daily rhythm in plasma glucagon concentrations in the rat is modulated by the biological clock and by feeding behavior. Diabetes 52, 1709–1715. doi: 10.2337/diabetes.52.7.1709, PMID: 12829637

[ref70] Sanchez D. I. Gonzalez-Fernandez B. Crespo I. San-Miguel B. Alvarez M. Gonzalez-Gallego J.. (2018). Melatonin modulates dysregulated circadian clocks in mice with diethylnitrosamine-induced hepatocellular carcinoma. J. Pineal Res. 65:e12506. doi: 10.1111/jpi.12506 29770483

[ref71] Sateia M. J. (2014). International classification of sleep disorders-third edition: highlights and modifications. Chest 146, 1387–1394. doi: 10.1378/chest.14-0970 25367475

[ref72] Schrader H. Bovim G. Sand T. (1993). The prevalence of delayed and advanced sleep phase syndromes. J. Sleep Res. 2, 51–55. doi: 10.1111/j.1365-2869.1993.tb00061.x 10607071

[ref73] Shouse M. N. Staba R. J. Saquib S. F. Farber P. R. (2000). Monoamines and sleep: microdialysis findings in pons and amygdala. Brain Res. 860, 181–189. doi: 10.1016/S0006-8993(00)02013-8, PMID: 10727641

[ref74] Shu Y. Yang Y. H. Xi Q. H. Hu J. Yao Y. (2021). Effects of simulated acupuncture on myocardial circadian clock genes Bmal1, clock and Per2 in spontaneously hypertensive rats. Acad. J. Shanghai Univ. Tradit. Chin. Med. 35, 89–93. doi: 10.16306/j.1008-861x.2021.04.013

[ref75] Socaciu A. I. Ionut R. Socaciu M. A. Ungur A. P. Barsan M. Chiorean A.. (2020). Melatonin, an ubiquitous metabolic regulator: functions, mechanisms and effects on circadian disruption and degenerative diseases. Rev. Endocr. Metab. Disord. 21, 465–478. doi: 10.1007/s11154-020-09570-9, PMID: 32691289

[ref76] Song X. J. Zhu Y. H. Wu P. Du L. Li Z. W. (2022). Acupoint compatibility effect and mechanism of Shenmen (HT7) and Sanyinjiao (SP6) in improving daytime fatigue and sleepiness of insomnia. Zhen Ci Yan Jiu 47, 630–635. doi: 10.13702/j.1000-0607.20210590, PMID: 35880281

[ref77] Spence D. W. Kayumov L. Chen A. Lowe A. Jain U. Katzman M. A.. (2004). Acupuncture increases nocturnal melatonin secretion and reduces insomnia and anxiety: a preliminary report. J. Neuropsychiatry Clin. Neurosci. 16, 19–28. doi: 10.1176/jnp.16.1.19, PMID: 14990755

[ref78] Suyama S. Yada T. (2018). New insight into GABAergic neurons in the hypothalamic feeding regulation. J. Physiol. Sci. 68, 717–722. doi: 10.1007/s12576-018-0622-8, PMID: 30003408 PMC10717766

[ref79] Tahara Y. Shibata S. (2018). Entrainment of the mouse circadian clock: effects of stress, exercise, and nutrition. Free Radic. Biol. Med. 119, 129–138. doi: 10.1016/j.freeradbiomed.2017.12.026, PMID: 29277444

[ref80] Takahashi J. S. (2015). Molecular components of the circadian clock in mammals. Diabetes Obes. Metab. 17, 6–11. doi: 10.1111/dom.12514, PMID: 26332962 PMC4560116

[ref81] Takahashi J. S. Hong H. K. Ko C. H. Mcdearmon E. L. (2008). The genetics of mammalian circadian order and disorder: implications for physiology and disease. Nat. Rev. Genet. 9, 764–775. doi: 10.1038/nrg2430, PMID: 18802415 PMC3758473

[ref82] Tan Q. Li B. C. Li J. Li J. Xiang H. C. Cai G. W. (2022). Acupuncture combined with moxibustion regulates the expression of circadian clock protein in the synovium of rats with osteoarthritis. Chin. J. Tissue Eng. Res. 26, 1714–1719.

[ref83] Tao Y. Liu J. N. Cai D. J. Zhao J. L. Zhou Q. Z. Wei J. L. (2014a). The effects of electric-acupuncture on the prokineticin2 of the suprachiasmatic nucleus in the rats under advances of light /dark cycle. Lishizhen Med. Mater. Med. Res. 25, 2295–2298. doi: 10.3969/j.issn.1008-0805.2014.09.107

[ref84] Tao Y. Zhang P. Liu J. N. Zhao J. L. Zhou Q. Z. Wei J. L.. (2014b). Effects of electric-acupuncture on the melatonin concentrations of blood plasma in the rats after the transfer of advanced light-dark cycle. Sichuan Zhongyi 32, 56–59. doi: CNKI:SUN:SZGY.0.2014-09-107

[ref85] Tsukamoto-Yamauchi N. Terasaka T. Iwasaki Y. Otsuka F. (2015). Interaction of pituitary hormones and expression of clock genes modulated by bone morphogenetic protein-4 and melatonin. Biochem. Biophys. Res. Commun. 459, 172–177. doi: 10.1016/j.bbrc.2015.02.100, PMID: 25727018

[ref86] Wang J. Chen Y. Zhai X. Chu Y. Liu X. Ma X. (2022). Visualizing research trends and identifying hotspots of traditional Chinese medicine (TCM) nursing Technology for Insomnia: a 18-years bibliometric analysis of web of science Core collection. Front. Neurol. 13:816031. doi: 10.3389/fneur.2022.816031, PMID: 35432182 PMC9009417

[ref87] Wang Z. H. Liu J. Guo B. J. Yu S. Y. Mao D. D. Shen Z. F.. (2016). Effect of acupuncture based on Qiao Meridian theory intervention on locomotor activity circadian rhythm in insomnia rat model. Liaoning J. Tradit. Chin. Med. 43, 2645–2648. doi: 10.13192/j.issn.1000-1719.2016.12.061

[ref88] Wang C. Sun S. M. Xiu Y. Wang Y. B. Miao J. R. Fan X. (2023). Effect of Electroacupuncture on clock and Bmal1 gene expressions in liver of C57BL /6J mice with circadian rhythm. Chin. Arch. Tradit. Chin. Med. 41, 243–248. doi: 10.13193/j.issn.1673-7717.2023.08.051

[ref89] Wang C. Xu W. L. Li G. W. Fu C. Li J. J. Wang J.. (2021). Impact of acupuncture on sleep and comorbid symptoms for chronic insomnia: a randomized clinical trial. Nat. Sci. Sleep 13, 1807–1822. doi: 10.2147/NSS.S326762 34675728 PMC8519353

[ref90] Wei X. R. Wei G. W. Zheng X. N. Wu X. F. Chen X. L. Liu L.. (2017). Effect of acupuncture stimulation of different Acupoint combinations on sleep and expression of circadian clock and Bmal 1 genes in hypothalamus of insomnia rats. Zhen Ci Yan Jiu 42, 429–433. doi: 10.13702/j.1000-0607.2017.05.010 PMID: 29105472

[ref91] Welsh D. K. Yoo S. H. Liu A. C. Takahashi J. S. Kay S. A. (2004). Bioluminescence imaging of individual fibroblasts reveals persistent, independently phased circadian rhythms of clock gene expression. Curr. Biol. 14, 2289–2295. doi: 10.1016/j.cub.2004.11.057, PMID: 15620658 PMC3777438

[ref92] Wu J. H. Chen H. Y. Chang Y. J. Wu H. C. Chang W. D. Chu Y. J.. (2009). Study of autonomic nervous activity of night shift workers treated with laser acupuncture. Photomed. Laser Surg. 27, 273–279. doi: 10.1089/pho.2007.2235, PMID: 18785846

[ref93] Xie C. Wang J. Zhao N. Yang W. Gao X. Liu Z.. (2021). Effects of Electroacupuncture on sleep via the dopamine system of the HPA Axis in rats after cage change. Evid. Based Complement. Alternat. Med. 2021, 1–25. doi: 10.1155/2021/5527060 PMC8270700 34306138

[ref94] Xie L. N. Xie Z. Q. Guo X. Wei X. R. Yue Z. H. (2018). Effects of acupuncture treatment based on meridians and Acupoint selection on MT content and MT receptor expressions in VLPO in insomnia rats. Chin. J. Inf. TCM 25, 40–44. doi: 10.3969/j.issn.1005-5304.2018.12.011

[ref95] Xiu Y. Wang Y. B. Wang C. Fan X. (2021). Acupuncture at Ganshu (BL18) regulates expressions of Cry1 and Cry2 in liver of mice with sleep-wake cycle disorder. Chin. Arch. Tradit. Chin. Med. 39, 203–208. doi: 10.13193/j.issn.1673-7717.2021.04.052

[ref96] Xu W. L. Wang C. Li J. J. Wang J. Chen Y. F. Liu Z. (2023). The effect of acupuncture on the sleep-wake circadian rhythm and pineal AANAT in model rats. Inf. Tradit. Chin. Med. 40, 1–8. doi: 10.19656/j.cnki.1002-2406.20230301

[ref97] Xue H. Zhu B. Wei J. L. Yin H. Y. Cai D. J. (2010). Adjusting effect of electro acupuncture point of Bai hui (GV20) and Chang Oiang (GV1) on heart rate rhythm of rats under phase inversion. J. Zhejiang Univ. Tradit. Chin. Med. 34, 893–896. doi: 10.16466/j.issn1005-5509.2010.06.018

[ref98] Yamazaki S. Numano R. Abe M. Hida A. Takahashi R. Ueda M.. (2000). Resetting central and peripheral circadian oscillators in transgenic rats. Science 288, 682–685. doi: 10.1126/science.288.5466.682, PMID: 10784453

[ref99] Yang H. Yang K. Zhang L. Yang N. Mei Y. X. Zheng Y. L.. (2023). Acupuncture ameliorates Mobile phone addiction with sleep disorders and restores salivary metabolites rhythm. Front. Psych. 14:1106100. doi: 10.3389/fpsyt.2023.1106100, PMID: 36896350 PMC9989025

[ref100] Ye Z. Zhu L. Li X. J. Gao H. Y. Wang J. Wu S. B.. (2023). PC6 electroacupuncture reduces stress-induced autonomic and neuroendocrine responses in rats. Heliyon 9:e15291. doi: 10.1016/j.heliyon.2023.e15291, PMID: 37095918 PMC10121450

[ref101] Yeung W. F. Chung K. F. Zhang S. P. Yap T. G. Law A. C. (2009). Electroacupuncture for primary insomnia: a randomized controlled trial. Sleep 32, 1039–1047. doi: 10.1093/sleep/32.8.1039, PMID: 19725255 PMC2717194

[ref102] Yu Q. Guo Q. Jin S. Gao C. Zheng P. Li D. P.. (2023). Melatonin suppresses sympathetic vasomotor tone through enhancing GABA(a) receptor activity in the hypothalamus. Front. Physiol. 14:1166246. doi: 10.3389/fphys.2023.1166246, PMID: 37064887 PMC10090494

[ref103] Yu X. T. Yang W. J. Zhao N. Liang R. L. Sun X. Q. Bi Y. P.. (2023). Acupuncture for delayed sleep-wake phase disorder: a randomized controlled trial. Zhongguo Zhen Jiu 43, 245–251. doi: 10.13703/j.0255-2930.20221031-k0002, PMID: 36858383

[ref104] Zhang F. Zhang X. Peng Q. Tang L. (2023). Electroacupuncture of the cymba concha alleviates p-chlorophenylalanine-induced insomnia in mice. Acupunct. Med. 41, 345–353. doi: 10.1177/09645284231160193 37081732

[ref105] Zylka M. J. Shearman L. P. Weaver D. R. Reppert S. M. (1998). Three period homologs in mammals: differential light responses in the suprachiasmatic circadian clock and oscillating transcripts outside of brain. Neuron 20, 1103–1110. doi: 10.1016/S0896-6273(00)80492-4, PMID: 9655499

